# COVID-19 Pandemic and Evolution of Telemedicine to TeleIME

**DOI:** 10.1080/20961790.2021.1895411

**Published:** 2021-03-29

**Authors:** Mohammed Ranavaya, Duarte Nuno Vieira

**Affiliations:** Division of Occupational, Environmental and Disability Medicine, Joan C. Edwards School of Medicine, Marshall University, Huntington, USA; Faculty of Medicine, University of Coimbra, Coimbra, Portugal; Institute of Legal Medicine and Institute of Bioethics, Coimbra, Portugal

The COVID-19 pandemic has been a life altering experience that very few could have predicted. During these unprecedented and uncertain times, with the majority of the world in some type of lockdown and travel restrictions in most places, medicine like many other fields of work has adapted to this challenging new normal. In particular the medical field has encouraged telemedicine and many patient visits have gone virtual.

Telemedicine, using telecommunication technology to exchange medical information and provide remote clinical care, arose in the 1960s to provide clinical examination and mental health support for patients at a distance [1]. The current pandemic has caused a need for remote learning, work, and meetings, and has led to robust videoconferencing technologies becoming widely available through commercial and social digital platforms. Use of smart phones, computers and tablets is already widespread in the US, with 90% of the adults using the Internet regularly [2]. Telemedicine has spread by leveraging the recent increased access to digital technology by all segments of the population in the recent years with the need for virtual visits by video mobile health applications [3]. The dramatic increase in the use of telemedicine during the pandemic is not only here to stay, but has found its use beyond its origi­nal mission of providing remote patient care [4].

The Independent Medical Examination (IME) and impairment and disability assessment performed remotely, dubbed by these authors as TeleIME, is a natural extension of traditional telemedicine. IMEs are a legally mandated medical examination for adjudication of workers’ compensation and other personal injury claims, as well as disability benefits under Social Security and other systems not only in the US but throughout the world. Pre-COVID, these examinations were performed in person, but given the risk of SARS-CoV-2 transmission from these in-person encounters and the elective nature of these examinations, there have been calls for canceling and/or delaying them. Examinees are either unable or unwilling to attend an IME due to morbidity and mortality from COVID, and many have been advised by their attorneys to reschedule for a later date due to the COVID-19 pandemic.

Since many SARS-CoV-2 infected individuals are asymptomatic and at this point there is no suitable option to screen for COVID-19 in the IME setting, the risks involved in an in-person IME for both the doctor and the examinee include, but are not limited to, asymptomatic virus shedding and infection of others, age and comorbidities of the parties involved, required travel, and IMEs being a “medically non- urgent, non-essential visit”. This has led some to question, should IME exams in person even be required during the current pandemic?

Enters the TeleIME and its many benefits and drawbacks. Many workers’ compensation systems, insurers and payors around the globe are now relying more heavily on TeleIME services to continue to process claims while also preserving the safety of healthcare professionals and patients [5].

Some IMEs, such as psychiatric evaluations, can be performed with relative confidence using teleconferencing platforms conducting psychological tests and mental status assessments. Similarly, dermatology exams can be done by inspection of skin lesions over video. However, others can present varying degrees of challenges. Given the frequent presentation of claimants with musculoskeletal complaints, it is important to have a framework for the virtual musculoskeletal physical examination [6]. This raises the related questions of how do you perform a *TeleIME*?

## The challenges of TeleIME

### Logistics issues

While under the current relaxed privacy laws necessitated by this pandemic, telehealth can be done by a physician in his or her office or home, communicating with the patient at home on a personal device with commonly available applications such as FaceTime, Zoom, Teams and others. However, unlike a medical encounter which may be relatively shorter than an IME, more sophisticated equipment at the claimant’s end would be required. The digital device’s hardware and software capability, the Internet connection and speed, particularly in rural communities where many of the claimants reside, may be significant challenges.

While a good history can be obtained during TeleIME, a physical examination cannot be done in a claimant’s house remotely. It would require a healthcare provider such as a physician assistant or a nurse practitioner (not possible by law in some countries) to perform a surrogate physical examination for the IME provider which will need a clai­mant to be at some sort of examination facility which would raise the same challenges and risks involved with an in-person IME.

For musculoskeletal examinations, a recent Mayo Clinic publication provides the medical practitioner a road map of performing a virtual musculoskeletal examination with a specific set of guidelines, both written and visual, to enhance the information obtained when evaluating the shoulder, hip, knee, ankle, and cervical and lumbar spine. In addition to photographs, accompanying videos are included to facilitate and demonstrate specific physical examination techniques that the patient can self-perform [7]. However, in a personal injury claim environment, these subject effort dependent examinations can be hard to reconcile unless a trained observer is present in the room to validate the effort. Moreover, the musculoskeletal examination, which includes among other things tasks such as range of motion measurement, manual muscle testing, deep tendon reflexes, tenderness and spasm, etc., can only be determined by an in-person physical examination. These challenges illustrate the current struggle and technological limitations in receiving the whole picture during a TeleIME.

So, if an IME provider was to perform an IME with the help of an assistant in a remote location, that person would have to be trained in at least some of the methodologies of evaluation of permanent impairment (namely the *AMA Guides to the Evaluation of Permanent Impairment (Guides)* [8]).

Additionally, there are specialized IMEs that would require organ function testing, e.g. pulmonary function test, exercise stress tests and imaging studies that cannot be done remotely and for which the claimant must travel to a suitable facility which can perform these tests.

### Legal and administrative issues

While virtual patient visits, i.e. telemedicine, during this pandemic have become the norm of the day in an attempt to minimize physical proximity between patient and healthcare staff and its attendant social interaction, performing *TeleIME* has its unique challenges. For instance, in a clinical setting with an established patient, one has a good knowledge of the patient’s medical background and history as opposed to an IME that in many instances is a component of some litigation. This is often an adversarial process and may require an examination in person to determine the sincerity of the effort on many aspects of physical examination.

Furthermore, many types of IME require direct in-person and hands-on collection of data which will be absent in TeleIME and would create signifi­cant potential for controversy and litigation. Anyone providing an opinion based on TeleIME may find oneself exposed to a lot of potential legal challenges and subject to vigorous cross-examination where a litany of issues could be brought up.

For example, there may be legal evidentiary objections to an expert opinion rendered based on the TeleIME because the doctor was not physically present during the examination. The relevancy and validity of such opinions could easily be challenged based on a lack of sufficient data and absence of reliable methodology. Either side of the Bar, unhappy with the conclusion of such an IME, would voci­ferously object to the admission of such evidence and attempt to pick apart findings based on reliance on an examination conducted by someone else, or worse, without a trained person in the room. The very accuracy of conclusions based on such exami­nation could be brought into question.

While the IME providers in the usual course of business quite often find their IME conclusions and opinions under attack by one side or the other, even in the in-person IME situation, the TeleIME would expose them to novel attacks which could include relying on hearsay and relying on physical exami­nation data obtained by someone else. There are defenses to that if one has a trained healthcare provider on the other side with the claimant. After all, in our daily healthcare work we all routinely rely on and draw diagnostic conclusions based on medi­cal information provided by our trained colleagues without necessarily risking the questioning of the wisdom of such an action. However, it is unclear at this point whether the judges would allow the same deference to the TeleIME and accept an exception to the hearsay rule for it.

While *AMA Guides to the Evaluation of Permanent Impairment* (*Guides*), as others similar guides (namely the European Guides—Guide barème européen d’évaluation médicale des atteintes à l’intégrité physique et psychique [9]), do not directly address the issue of TeleIME as it could not have been contemplated when the guides were constructed, in multiple places the *Guides* do emphasize that the examiner must assess the reliability of vari­ous components of the IME. This includes the functional limitation reports by the claimant, recog­nizing the potential influence of behavioural and psychosocial factors.

*Guides* further emphasize that inconsistencies between the claimant’s subjective complaints, reported functional loss and the IME providers in office objective findings and observation of the claimant is criti­cal to arriving at a correct impairment rating. This could arguably be absent in a TeleIME situation. Some attorneys, unhappy with the outcome of an IME, would certainly make a case to the court to exclude the evidence based on such TeleIME as the entire process weakens the database upon which the foundation of an IME opinion lies.

With the TeleIME, the entire encounter could be recorded with a potential for cherry picking by opposing counsel on cross-examination. The surrogate physical examiner could also be subject to subpoena and cross-examination and their knowledge, skills, abilities and training could also be brought into question, necessitating certifications of such personnel, and the absence of which could raise questions about the very foundation upon which an IME provider has formed her opinions and conclusions.

TeleIME may further tempt some providers to reach across jurisdictions and provide IME services across state lines which may produce its own legal problems, namely issues of medical licensing in a state where the claimant resides. This may not be a problem in countries where there is a countrywide medical registration process with practitioners free to practice anywhere in the country. However, in the US medical licensing is a state jurisdiction and this must be remembered before reaching out into other jurisdictions and providing IME opinions without a license to practice [10].

TeleIME may not be a universally acceptable method for workers’ compensation and other disa­bility management systems, particularly in an adversarial litigation process. Similar situations may occur in other parts of the world. There remain significant legal issues in regards to implementation of TeleIME, and only the competent legal authorities will be the final arbiters.

## Is TeleIME dead on arrival?

The answer is no. TeleIME can be done if accepted by all stakeholders. Not all IMEs are the same. For example, pulmonary IMEs can be done remotely if there is a trained healthcare provider at the other end that can perform the necessary chest examination, including auscultation and providing the findings. Additionally, the pulmonary function studies and imaging studies can be done at the hospital or clinic. This would still require a claimant to travel to a facility and be at least examined by a healthcare provider, so it will not be a true TeleIME but an experienced pulmonary expert can do this remotely with the help of a physician extender performing the necessary physical examination.

Similarly, psychiatric IMEs are done remotely in some parts of the world. For example, over the past several years, Australian IME psychiatrists have been performing IMEs on secured telehealth portals. They have had to do this because some claimants are located in the sparsely populated areas of the country and lack expert psychiatrists in remote parts of Australia. They have been quite successful in getting psych IMEs accepted when performed *via* video links as psychiatrists do not always have to perform a physical examination. This allows the IME to be done at any place of convenience for the claimant, including one’s home, if a suitable and secure Internet connected device is available. The issue of getting specific psychiatric testing has also been resolved by our Australian colleagues in a very innovative way.

Other than the limited potential for TeleIME as described above, significant challenges remain both legal and technical for TeleIME to replace the traditional in-person IME any time soon. There are, however, alternatives that can answer many questions found on a typical IME without actually performing one. Many questions can be answered if sufficient and adequate medical record is available to the IME provider. In fact, many IME referral sources during this pandemic are opting for records reviews alone in order to settle a claim. After all, 95% to 97% of all civil actions are resolved by settlement. Many routine IME issues such as causation, work relatedness, treatment necessity, and return to work can be answered based on record and without performing an IME. In some cases, a record review and opinion to various issues can be reasonably provided with the hope that a settlement would occur, failure of which can trigger an IME at a later date when it is safe to do so in person.

These are unprecedented times and require innovative solutions. This greater use of medical record reviews in a virtual environment would certainly require knowledge, skills and technical know-how that many medicolegal experts and IME providers lack at this time.

## Final thoughts

In summary, the current scientific evidence suggests that COVID-19 is not going to suddenly disappear any time soon. It is probably going to stalk humanity for far longer than any of us wish, and may have changed our lives forever. This means that going forward the IME community will have to adapt and learn to live with it. Even as vaccination produces increased immunity, the pandemic eases, and the restrictions lift, change will be gradual. We will be seeing social distancing, facemasks and other rigorous precautions in place for some time to come as the IME community tries to establish some semblance of normalcy. Until our social environment returns to normal, in-person IME demands by insurance companies or other referral sources may raise seemingly legiti­mate questions as to whether some persons with risk factors such as age and comorbidity should be mandated to go for an IME that exposes the claimant to dangers such as a specialist’s crowded waiting room where maintaining a “social distance” from others is challenging, or worse, in large multi-specialty group practices within larger facilities with common waiting areas full of different types of patients. There may be potential liability concerns if these claimants are forced to attend in-person IMEs without proper protection.

In-person IMEs are preferred and, in some instances, indispensable for resolving certain issues, however at this juncture it appears that at least in the near future there will be a signifi­cant decline in face-to-face IMEs. TeleIMEs and robust file and record reviews may be an accepta­ble alternative in some circumstances. Significant improvement in technology and wider legal acceptance of TeleIME would make virtual IME visits the norm rather than the novelty they currently remain.
